# Prognostic significance of vitamin D receptor (*VDR*) gene polymorphisms in liver cirrhosis

**DOI:** 10.1038/s41598-018-32482-3

**Published:** 2018-09-14

**Authors:** Christos Triantos, Ioanna Aggeletopoulou, Maria Kalafateli, Panagiota I. Spantidea, Georgia Vourli, Georgia Diamantopoulou, Dimitra Tapratzi, Marina Michalaki, Spilios Manolakopoulos, Charalambos Gogos, Venetsana Kyriazopoulou, Athanasia Mouzaki, Konstantinos Thomopoulos

**Affiliations:** 1 Department of Gastroenterology, University Hospital of Patras, Patras, Greece; 2 Division of Hematology, Department of Internal Medicine, Medical School, University of Patras, Patras, Greece; 3 Department of Hygiene, Epidemiology and Medical Statistics, Medical School, University of Athens, Athens, Greece; 4 Division of Endocrinology, Diabetes and Metabolic Diseases, Department of Internal Medicine, University of Patras, Patras, Greece; 5 2nd Department of Internal Medicine, Hippokration General Hospital of Athens, 11527 Athens, Greece; 6 Department of Internal Medicine, University Hospital of Patras, Patras, Greece

## Abstract

Several polymorphisms in the vitamin D receptor (*VDR*) are associated with the occurrence of chronic liver disease. Here, we investigated the association between BsmI, ApaI, TaqI and FokI *VDR* polymorphisms and the severity of liver cirrhosis in relation to serum cytokine and lipopolysaccharide binding protein (LBP) levels and their role on survival in cirrhotic patients. We found that patients harboring the BB genotype had higher MELD score, and they were mainly at CP stage C; patients harboring the AA genotype had increased LBP, IL-1β and IL-8 levels, and they were mostly at CP stage C; TT genotype carriers had higher MELD score and they were mainly at CP stage C and FF genotype carriers had lower IL-1β levels when compared to Bb/bb, Aa/aa, Tt/tt and Ff/ff genotypes respectively. In the multivariate analysis ApaI, BsmI and TaqI polymorphisms were independently associated with liver cirrhosis severity. In the survival analysis, the independent prognostic factors were CP score, MELD and the FF genotype. Our results indicate that the ApaI, TaqI and BsmI polymorphisms are associated with the severity of liver cirrhosis, through the immunoregulatory process. Survival is related to the FF genotype of FokI polymorphism, imparting a possible protective role in liver cirrhosis.

## Introduction

Liver cirrhosis is defined as the histological development of regenerative nodules surrounded by fibrous bands in response to chronic liver injury, and is associated with the development of liver failure and portal hypertension^1,2^. Infection with Hepatitis B (HBV) or C (HCV), alcohol abuse and nonalcoholic fatty liver disease (NAFLD) are the main etiologic factors of liver cirrhosis worldwide^1,2^. However, certain genetic polymorphisms may influence the progression of liver fibrosis^3^.

The vitamin D receptor (*VDR*) is a DNA-binding transcription factor that is expressed on peripheral blood (PB) monocytes and activated T lymphocytes. *VDR* belongs to the nuclear receptor superfamily and is associated with many physiological processes^4–6^. The most common polymorphisms of the *VDR* gene are the BsmI, FokI, TaqI and ApaI. FokI, is located in exon 2 of the *VDR* gene and the presence of this polymorphism results in a shortened VDR protein due to an alteration in the start codon^7^. The ApaI and the BsmI polymorphisms are located in intron 8 at the 3′ end of the *VDR* gene. These polymorphisms do not change the amino acid sequence of the VDR protein. However, BsmI and ApaI may affect gene expression through the alteration of mRNA stability, the disruption of splice sites for mRNA transcription, or a change in intronic regulatory elements^8,9^. The TaqI polymorphism is located in exon 9 at the 3′ end of the human *VDR* gene and results in a synonymous change due to a nucleotide substitution. The presence of TaqI polymorphism does not modify the VDR protein but is involved in the regulation of the stability of *VDR* mRNA^8,9^. Recent studies have shown that there is a genetic association of *VDR* polymorphisms to autoimmune hepatitis (AIH), primary biliary cirrhosis (PBC), HBV infection and hepatocellular carcinoma (HCC)^8,10–17^. Moreover, the progression of liver fibrosis has been associated with the existence of *VDR* polymorphisms in patients with PBC^10^ and HCV^18^ and with reduced full-length VDR protein expression, but increased VDR protein fragments in patients with NAFLD^10,18,19^.

Cytokines are key mediators in the pathophysiology of liver disease as they play an essential role in hepatic regeneration and fibrosis^20^. The hepatic non parenchymal cells which are involved in liver fibrosis development, can rapidly produce profibrogenic cytokines which lead to hepatic inflammation and fibrosis^21^. In contrast, antifibrogenic cytokines downregulate the pro-inflammatory response promoting the hepatic regeneration^20,21^. *VDR* polymorphisms may influence the immune regulation by affecting cytokine levels and, thus, they might play a role in the progression of liver disease^11,13^.

In this study, we have investigated the potential associations between *VDR* gene polymorphisms and the severity of liver cirrhosis, in relation to the cytokine and bacterial profiles, vitamin D and vitamin D binding protein (VDBP) levels, and their role on patient survival.

## Results

The main demographic and clinical characteristics of the examined patients are presented in Table 1 and the main characteristics of the examined *VDR* polymorphisms are presented in Table 2.

**Table 1:** Patients’ demographic and main clinical baseline characteristics.

	Mean	Range
Age (years)	60.74	29–84
	**N**	**Percentage (%)**
Sex (M/F)	60/29	67.4/32.6
**Etiology of liver cirrhosis**	**N**	**Percentage (%)**
Alcohol consumption	32	36.4
HBV ± HDV infection	29	32.5
HCV infection	10	10.4
Cryptogenic cirrhosis	6	6.5
Autoimmune hepatitis	5	5.2
Primary biliary cirrhosis	3	3.9
Nonalcoholic steatohepatitis	2	2.6
Primary sclerosing cholangitis	1	1.3
HBV infection + HCV infection	1	1.3
	**Median (IQR)**	
Vitamin D levels (ng/mL)	21.1 (14.7, 31.6)	
Hb (g/dL)	12.7 (11.2, 13.8)	
Plt (K/uL)	117.5 (66, 160)	
INR	1.3 (1.1, 1.6)	
Creatinine (mg/dL)	0.9 (0.7, 1.1)	
SGPT (U/L)	31.0 (21.0, 58.0)	
G-GT (U/L)	54.0 (29.0, 109.0)	
ALP (U/L)	101.5 (76.0, 142.0)	
Albumin (g/dL)	3.6 (3.0, 4.0)	
Total Bilirubin (mg/dL)	1.3 (0.7, 2.5)	
K (mmol/L)	4.3 (3.9, 4.7)	
Na (mmol/L)	138.4 (135.2, 141.0)	
Ca (mmol/L)	9.0 (8.6, 9.4)	
Mg (mmol/L)	1.9 (1.6, 2.1)	
Systolic pressure (mmHg)	130.0 (115.0, 150.0)	
Diastolic pressure (mmHg)	75.0 (70.0, 80.0)	
CP score	6.0 (5.0, 9.0)	
MELD	11.0 (8.0, 15.5)	
VDBP (µg/mL)	160.8 (99.3, 257.9)	
IL-12 (pg/mL)	7.5 (2.1, 8.7)	
TNF-a (pg/mL)	4.7 (1.2, 5.7)	
IL-1β (pg/mL)	8.0 (3.1, 11.6)	
IL-6 (pg/mL)	7.8 (4.3, 22.0)	
IL-8 (pg/mL)	35.7 (20.6, 90.7)	
IL-10 (pg/mL)	3.9 (3.1, 4.7)	
LBP (μg/mL)	10.8 (8.3, 11.4)	

Abbreviations: M, male; F, female; HBV, hepatis B virus; HDV, hepatis D virus; HCV, hepatis C virus; IQR, Interquartile range; Hb, Hemoglobin; Plt, Platelets; INR, International normalized ratio; SGPT, Alanine aminotransferase; G-GT, Gamma-Glutamyl Transferase; ALP, Alkaline phosphatase; CP, Child Pugh; MELD, Model for end-stage liver disease; VDBP, Vitamin D binding protein; LBP, Lipopolysaccharide binding protein.

**Table 2:** Characterization of *VDR* polymorphisms.

SNP name	SNP ID	SNP location	Nucleotide change	Correspondence of nomenclature of SNP alleles
FokI	rs2228570	Exon 2	C > T	F > f
BsmI	rs1544410	Intron 8	A > G	B > b
TaqI	rs731236	Exon 9	C > T	T > t
ApaI	rs7975232	Intron 8	A > C	A > a

Abbreviations: SNP, single nucleotide polymorphism.

### Distribution of clinical variables and serum cytokine expression according to the *VDR* genotypes

As shown in Table 3 the presence of BsmI polymorphism, in particular the BB genotype, was associated with advanced Child-Pugh (CP) stage (*p* = 0.044) and higher model for the end-stage liver disease (MELD) score (*p* = 0.045). The AA genotype of the ApaI polymorphism was associated with advanced CP stage (*p* = 0.001) and increased LBP levels (*p* = 0.014). The presence of TaqI polymorphism (TT genotype) was associated with advanced CP stage (*p* = 0.027) and MELD score (*p* = 0.025). As regards to the FokI polymorphism, the FF genotype was associated with lower levels of the pro-inflammatory cytokine IL-1β (*p* = 0.045).

**Table 3:** Distribution of clinical variables according to the *VDR* genotypes.

BsmI A > G (rs1544410)					
	bb	Bb	BB	Overall	*p-*value
**N (%)**					
CP stage					**0.044**
A	12 (45.8)	32 (60.4)	2 (22.2)	46 (51.3)	
B	11 (41.7)	13 (23.3)	2 (22.2)	26 (28.9)	
C	3 (12.5)	9 (16.3)	5 (55.6)	17 (19.8)	
**Median (IQR)**					
Vitamin D levels (ng/mL)	20.6 (14.8, 29.6)	21.5 (14.7, 31.6)	29.8 (11.2, 46.7)	21.1 (14.7, 31.6)	0.828
MELD	12.0 (8.5, 17.0)	10.0 (8.0, 14.0)	16.0 (12.0, 19.0)	11.0 (8.0, 15.5)	**0.045**
VDBP (μg/mL)	177.6 (143.9, 259.0)	136.4 (89.7, 256.7)	199.3 (126.8, 209.6)	160.8 (99.3, 257.9)	0.307
IL-12 (pg/mL)	6.9 (0.0, 8.7)	8.0 (5.6, 8.7)	5.9 (0.0, 6.8)	7.5 (2.1, 8.7)	0.438
TNF-a (pg/mL)	4.9 (1.0, 5.9)	4.6 (1.5, 5.3)	5.2 (0.3, 6.0)	4.7 (1.2, 5.7)	0.764
IL-1β (pg/mL)	8.1 (6.5, 12.6)	7.9 (6.2, 11.5)	7.5 (0.0, 11.2)	8.0 (3.1, 11.6)	0.752
IL-6 (pg/mL)	5.8 (5.1, 12.8)	7.8 (4.3, 27.2)	13.0 (6.5, 49.4)	7.8 (4.3, 22.0)	0.520
IL-8 (pg/mL)	33.5 (16.0, 101.6)	41.3 (23.0, 87.9)	29.3 (20.9, 44.3)	35.7 (20.6, 90.7)	0.698
IL-10 (pg/mL)	3.7 (2.0, 4.2)	4.0 (3.3, 5.2)	4.0 (3.7, 6.3)	3.9 (3.1, 4.7)	0.257
LBP (μg/mL)	9.8 (4.9, 11.3)	10.8 (8.4, 11.6)	11.3 (11, 11.4)	10.8 (8.3, 11.4)	0.128
**FokI C** > **T (rs10735810)**					
	**ff**	**Ff**	**FF**	**Overall**	***p-*** **value**
**N (%)**					
CP stage					0.846
A	4 (42.8)	21 (53)	21 (51.4)	46 (51.3)	
B	4 (42.8)	9 (23.5)	13 (31.4)	26 (28.9)	
C	1 (14.2)	9 (23.5)	7 (17.2)	17 (19.8)	
**Median (IQR)**					
Vitamin D levels (ng/mL)	30.3 (21.3, 46.7)	22.0 (14.7, 32.2)	18.4 (13.9, 26.4)	21.1 (14.7, 31.6)	0.139
MELD	12.0 (9.0, 13.0)	11.0 (8.0, 16.0)	11.0 (8.0, 17.0)	11.0 (8.0, 15.5)	0.983
VDBP (μg/mL)	217.8 (189.0, 282.9)	147.3 (112.2, 270.4)	159.3 (89.7, 220.8)	160.8 (99.3, 257.9)	0.355
IL-12 (pg/mL)	5.3 (0.0, 8.2)	7.5 (5.3, 8.9)	8.0 (0.0, 8.5)	7.5 (2.1, 8.7)	0.382
TNF-a (pg/mL)	4.9 (1.6, 5.8)	4.6 (1.5, 5.7)	4.7 (0.4, 5.7)	4.7 (1.2, 5.7)	0.846
IL-1β (pg/mL)	9.3 (7.5, 11.8)	9.4 (7.4, 12.3)	7.2 (0.0, 10.1)	8.0 (3.1, 11.6)	**0.045**
IL-6 (pg/mL)	5.8 (5.1, 7.7)	7.8 (4.2, 20.1)	9.8 (4.9, 29.4)	7.8 (4.3, 22.0)	0.712
IL-8 (pg/mL)	33.7 (20.8, 87.9)	44.7 (22.6, 110.4)	33.0 (20.4, 85.8)	35.7 (20.6, 90.7)	0.812
IL-10 (pg/mL)	3.7 (1.8, 4.7)	3.8 (3.2, 4.6)	4.0 (3.2, 4.6)	3.9 (3.1, 4.7)	0.970
LBP (μg/mL)	10.8 (9.6, 11.3)	10.5 (8.7, 11.3)	10.9 (4.3, 11.4)	10.8 (8.3, 11.4)	0.995
**ApaI G** > **T (rs7975232)**					
	**aa**	**Aa**	**AA**	**Overall**	***p-*** **value**
**N (%)**					
CP stage					**0.001**
A	3 (30)	29 (63.4)	13 (40)	45 (51.3)	
B	7 (60)	14 (29.3)	5 (16)	26 (28.9)	
C	1 (10)	3 (7.3)	14 (44)	18 (19.8)	
**Median (IQR)**					
Vitamin D levels (ng/mL)	20.0 (9.5, 26.2)	20.8 (14.9, 30.4)	24.1 (14.1, 33.6)	21.1 (14.7, 31.6)	0.754
Diastolic pressure (mmHg)	60.0 (60.0, 70.0)	77.5 (70.0, 90.0)	77.5 (70.0, 80.0)	75.0 (70.0, 80.0)	**0.011**
MELD	9.5 (8.0, 14.0)	11.0 (8.0, 14.0)	13.0 (9.0, 17.0)	11.0 (8.0, 15.5)	0.377
VDBP (μg/mL)	244.9 (157.6, 288.6)	170.8 (101.1, 256.7)	130.2 (81.7, 215.3)	160.8 (99.3, 257.9)	0.140
IL-12 (pg/mL)	8.5 (5.1, 9.0)	7.7 (0.0, 8.5)	7.0 (4.7, 8.5)	7.5 (2.1, 8.7)	0.794
TNF-a (pg/mL)	5.6 (1.0, 5.9)	4.4 (1.5, 5.2)	5.0 (1.1, 6.1)	4.7 (1.2, 5.7)	0.394
IL-1β (pg/mL)	8.1 (0.0, 12.6)	7.3 (0.0, 9.6)	10.1 (7.5, 11.7)	8.0 (3.1, 11.6)	0.076
IL-6 (pg/mL)	7.7 (5.5, 12.3)	5.8 (4.1, 17.6)	12.9 (5.2, 29.8)	7.8 (4.3, 22.0)	0.177
IL-8 (pg/mL)	33.7 (19.9, 146.9)	27.9 (19.5, 83.5)	45.5 (24.4, 200.2)	35.7 (20.6, 90.7)	0.076
IL-10 (pg/mL)	3.7 (0.8, 4.0)	3.9 (3.1, 4.6)	4.1 (3.4, 5.4)	3.9 (3.1, 4.7)	0.297
LBP (μg/mL)	9.4 (3.8, 11.3)	10.2 (4.6, 11.3)	11.3 (10.5, 11.4)	10.8 (8.3, 11.4)	**0.014**
**TaqI C** > **T (rs731236)**					
	**tt**	**Tt**	**TT**	**Overall**	***p-*** **value**
**N (%)**					
CP stage					**0.027**
A	12 (42.3)	33 (63.4)	2 (22.2)	47 (51.3)	
B	12 (42.3)	11 (22)	2 (22.2)	25 (28.9)	
C	4 (15.4)	8 (14.6)	5 (55.6)	17 (19.8)	
**Median (IQR)**					
Vitamin D levels (ng/mL)	21.2 (15.5, 30.8)	20.8 (14.7, 30.3)	29.8 (11.2, 46.7)	21.1 (14.7, 31.6)	0.719
MELD	12.0 (9.0, 17.0)	10.0 (8.0, 14.0)	16.0 (12.0, 19.0)	11.0 (8.0, 15.5)	**0.025**
VDBP (μg/mL)	175.8 (123.8, 259.0)	140.5 (90.9, 259.3)	199.3 (126.8, 209.6)	160.8 (99.3, 257.9)	0.597
IL-12 (pg/mL)	8.0 (0.0, 9.0)	7.8 (5.4, 8.6)	5.9 (0.0, 6.8)	7.5 (2.1, 8.7)	0.475
TNF-a (pg/mL)	5.2 (1.0, 5.9)	4.5 (1.5, 5.3)	5.2 (0.3, 6.0)	4.7 (1.2, 5.7)	0.595
IL-1β (pg/mL)	8.1 (6.5, 12.6)	7.9 (3.1, 11.6)	7.5 (0.0, 11.2)	8.0 (3.1, 11.6)	0.786
IL-6 (pg/mL)	7.7 (5.1, 13.8)	7.6 (4.3, 23.6)	13.0 (6.5, 49.4)	7.8 (4.3, 22.0)	0.569
IL-8 (pg/mL)	33.7 (16.0, 101.6)	41.3 (22.9, 90.7)	29.3 (20.9, 44.3)	35.7 (20.6, 90.7)	0.759
IL-10 (pg/mL)	3.8 (2.0, 4.4)	3.9 (3.3, 5.0)	4.0 (3.7, 6.3)	3.9 (3.1, 4.7)	0.468
LBP (μg/mL)	10.2 (5.2, 11.3)	10.7 (8.2, 11.4)	11.3 (11.0, 11.4)	10.8 (8.3, 11.4)	0.200

Abbreviations: IQR, Interquartile range; CP, Child Pugh; MELD, Model for end-stage liver disease; VDBP, Vitamin D binding protein; LBP, Lipopolysaccharide binding protein.

### Comparisons of clinical parameters and serum cytokine expression between *VDR* polymorphisms

As shown in Table 4, BsmI patients harboring the BB genotype had higher MELD score (*p* = 0.026) and were mainly at CP stage C (*p* = 0.020) compared to Bb/bb genotypes. ApaI patients harboring the AA genotype had increased levels of LBP (*p* = 0.004), IL-1β (*p* = 0.036) and IL-8 (*p* = 0.03) and were mostly at CP stage C (*p* = 0.001) compared to patients with the Aa/aa genotypes. The TT genotype carriers of the TaqI polymorphism had higher MELD score (*p* = 0.026) and were mainly at CP stage C (*p* = 0.02). Finally, FokI patients who had the FF genotype showed lower levels of IL-1β (*p* = 0.013) compared to patients with the Ff/ff genotypes. In the multivariate analysis, in the presence of other significant covariates, as well as cirrhosis’ etiology, AA genotype of ApaI polymorphism [OR: 5.5; 95% CI (1.3, 22.9), *p* = 0.019], BB genotype of BsmI polymorphism [OR: 9.6; 95% CI (1.3, 72.20), *p* = 0.027] and TT genotype of TaqI polymorphism [OR: 9.6; 95% CI (1.3, 72.20), *p* = 0.027] were found to be associated with increased odds of more advanced CP stage when compared to Aa/aa, Bb/bb and Tt/tt genotypes respectively (Table 5). FF genotype of FokI polymorphism was not found to be a significant predictor of disease severity.

**Table 4:** Comparisons of clinical parameters and serum cytokine expression between *VDR* polymorphisms.

BsmI A > G (rs1544410)				
	Bb + bb	BB	Overall	*p-*value
**N (%)**				
CP stage				**0.020**
A	44 (55.3)	2 (22.2)	46 (51.3)	
B	24 (29.8)	2 (22.2)	26 (28.9)	
C	12 (14.9)	5 (55.6)	17 (19.8)	
**Median (IQR)**				
Vitamin D levels (ng/mL)	21.0 (14.8, 30.3)	29.8 (11.2, 46.7)	21.1 (14.7, 31.6)	0.540
VDBP (μg/mL)	157.6 (97.4, 259.0)	199.3 (126.8, 209.6)	160.8 (99.3, 257.9)	0.890
MELD	11.0 (8.0, 14.0)	16.0 (12.0, 19.0)	11.0 (8.0, 15.5)	**0.026**
IL-12 (pg/mL)	7.9 (5.1, 8.7)	5.9 (0.0, 6.8)	7.5 (2.1, 8.7)	0.225
TNF-a (pg/mL)	4.6 (1.3, 5.7)	5.2 (0.3, 6.0)	4.7 (1.2, 5.7)	0.804
IL-1β (pg/mL)	8.1 (6.2, 11.8)	7.5 (0.0, 11.2)	8.0 (3.1, 11.6)	0.593
IL-6 (pg/mL)	7.7 (4.3, 18.1)	13.0 (6.5, 49.4)	7.8 (4.3, 22.0)	0.298
IL-8 (pg/mL)	37.7 (20.4, 93.4)	29.3 (20.9, 44.3)	35.7 (20.6, 90.7)	0.778
IL-10 (pg/mL)	3.8 (3.1, 4.6)	4.0 (3.7, 6.3)	3.9 (3.1, 4.7)	0.449
LBP (μg/mL)	10.5 (7.0, 11.4)	11.3 (11.0, 11.4)	10.8 (8.3, 11.4)	0.121
**FokI C** > **T (rs10735810)**				
	**Ff + ff**	**FF**	**Overall**	***p-*** **value**
**N (%)**				
CP stage				0.829
A	25 (51.2)	21 (51.5)	46 (51.3)	
B	13 (26.8)	13 (31.4)	26 (28.9)	
C	10 (22)	7 (17.1)	17 (19.8)	
**Median (IQR)**				
Vitamin D levels (ng/mL)	22.8 (15.9, 33.3)	18.4 (13.9, 26.4)	21.1 (14.7, 31.6)	0.206
VDBP (μg/mL)	168.9 (113.7, 270.6)	159.3 (89.7, 220.8)	160.8 (99.3, 257.9)	0.309
MELD	11.0 (8.0, 15.0)	11.0 (8.0, 17.0)	11.0 (8.0, 15.5)	0.867
IL-12 (pg/mL)	7.0 (4.2, 8.9)	8.0 (0.0, 8.5)	7.5 (2.1, 8.7)	0.840
TNF-a (pg/mL)	4.6 (1.5, 5.7)	4.7 (0.4, 5.7)	4.7 (1.2, 5.7)	0.580
IL-1β (pg/mL)	9.3 (7.4, 12.3)	7.2 (0.0, 10.1)	8.0 (3.1, 11.6)	**0.013**
IL-6 (pg/mL)	7.1 (4.2, 17.6)	9.8 (4.9, 29.4)	7.8 (4.3, 22.0)	0.459
IL-8 (pg/mL)	38.1 (20.8, 110.4)	33.0 (20.4, 85.8)	35.7 (20.6, 90.7)	0.555
IL-10 (pg/mL)	3.8 (3.0, 4.7)	4.0 (3.1, 4.6)	3.9 (3.1, 4.7)	0.907
LBP (μg/mL)	10.5 (9.0, 11.3)	10.9 (4.3, 11.4)	10.8 (8.3, 11.4)	0.983
**ApaI A** > **C (rs7975232)**				
	**Aa** + **aa**	**AA**	**Overall**	***p-*** **value**
**N (%)**				
CP stage				**0.001**
A	32 (56.9)	13 (40)	45 (51.3)	
B	20 (35.3)	5 (16)	25 (28.9)	
C	5 (7.8)	14 (44)	19 (19.8)	
**Median (IQR)**				
Vitamin D levels (ng/mL)	20.6 (14.8, 30.3)	24.1 (14.1, 33.6)	21.1 (14.7, 31.6)	0.527
VDBP (μg/mL)	178.9 (123.4, 260.4)	130.2 (81.7, 215.3)	160.8 (99.3, 257.9)	0.161
MELD	11.0 (8.0, 14.0)	13.0 (9.0, 17.0)	11.0 (8.0, 15.5)	0.167
IL-12 (pg/mL)	7.8 (0.0, 8.7)	7.0 (4.7, 8.5)	7.5 (2.1, 8.7)	0.704
TNF-a (pg/mL)	4.5 (1.3, 5.6)	5.0 (1.1, 6.1)	4.7 (1.2, 5.7)	0.258
IL-1β (pg/mL)	7.5 (0.0, 11.4)	10.1 (7.5, 11.7)	8.0 (3.1, 11.6)	**0.036**
IL-6 (pg/mL)	6.5 (4.1, 13.3)	12.9 (5.2, 29.8)	7.8 (4.3, 22.0)	0.063
IL-8 (pg/mL)	29.8 (19.6, 84.4)	45.5 (24.4, 200.2)	35.7 (20.6, 90.7)	**0.030**
IL-10 (pg/mL)	3.8 (3.0, 4.5)	4.1 (3.5, 5.5)	3.9 (3.1, 4.7)	0.221
LBP (μg/mL)	9.9 (4.8, 11.3)	11.3 (10.5, 11.4)	10.8 (8.3, 11.4)	**0.004**
**TaqI C** > **T (rs731236)**				
	**Tt + tt**	**TT**	**Overall**	***p-*** **value**
**N (%)**				
CP stage				**0.020**
A	44 (55.3)	2 (22.2)	46 (51.3)	
B	24 (29.8)	2 (22.2)	26 (28.9)	
C	12 (14.9)	5 (55.6)	17 (19.8)	
**Median (IQR)**				
Vitamin D levels (ng/mL)	21.0 (14.8, 30.3)	29.8 (11.2, 46.7)	21.1 (14.7, 31.6)	0.540
VDBP (μg/mL)	157.6 (97.4, 259.0)	199.3 (126.8, 209.6)	160.8 (99.3, 257.9)	0.890
MELD	11.0 (8.0, 14.0)	16.0 (12.0, 19.0)	11.0 (8.0, 15.5)	**0.026**
IL-12 (pg/mL)	7.9 (5.1, 8.7)	5.9 (0.0, 6.8)	7.5 (2.1, 8.7)	0.225
TNF-a (pg/mL)	4.6 (1.3, 5.7)	5.2 (0.3, 6.0)	4.7 (1.2, 5.7)	0.804
IL-1β (pg/mL)	8.1 (6.2, 11.8)	7.5 (0.0, 11.2)	8.0 (3.1, 11.6)	0.593
IL-6 (pg/mL)	7.7 (4.3, 18.1)	13.0 (6.5, 49.4)	7.8 (4.3, 22.0)	0.298
IL-8 (pg/mL)	37.7 (20.4, 93.4)	29.3 (20.9, 44.3)	35.7 (20.6, 90.7)	0.778
IL-10 (pg/mL)	3.8 (3.1, 4.6)	4.0 (3.7, 6.3)	3.9 (3.1, 4.7)	0.449
LBP (μg/mL)	10.5 (7.0, 11.4)	11.3 (11.0, 11.43)	10.8 (8.3, 11.4)	0.121

Abbreviations: IQR, Interquartile range; CP, Child Pugh; MELD, Model for end-stage liver disease; VDBP, Vitamin D binding protein; LBP, Lipopolysaccharide binding protein.

**Table 5:** Multivariate analysis for the association of VDR polymorphisms with cirrhosis severity by means of CP stage.

Factor	*OR*	95% C.I.	*p*-value
SNP_ApaI			
Aa + aa*	1		
AA	5.51	(1.33, 22.89)	0.019
Etiology			
alcohol^*^	1		
viral	5.01	(1.11, 22.71)	0.036
other	11.74	(1.34, 102.94)	0.026
Sex			
male*	1		
female	0.10	(0.02, 0.53)	0.007
VDBP			
per unit	0.98	(0.97, 0.99)	<0.001
IL-8			
per unit	1.01	(1.00, 1.02)	0.005
LBP			
per unit	1.02	(1.00, 1.05)	0.031
SNP_BsmI			
Bb + bb*	1		
BB	9.64	(1.29, 72.20)	0.027
Etiology			
alcohol*	1		
viral	3.66	(0.89, 15.05)	0.072
other	7.78	(0.94, 64.15)	0.057
Sex			
male*	1		
female	0.12	(0.02, 0.62)	0.012
VDBP			
per unit	0.98	(0.97, 0.99)	<0.001
IL-8			
per unit	1.01	(1.00, 1.02)	0.003
LBP			
per unit	1.02	(1.00, 1.05)	0.031
SNP_TaqI			
Tt + tt*	1		
TT	9.64	(1.29, 72.20)	0.027
Etiology			
alcohol*	1		
viral	3.66	(0.89, 15.05)	0.072
other	7.78	(0.94, 64.15)	0.057
Sex			
male*	1		
female	0.12	(0.02, 0.62)	0.012
VDBP			
per unit	0.98	(0.97, 0.99)	<0.001
IL-8			
per unit	1.01	(1.00, 1.02)	0.003
LBP			
per unit	1.02	(1.00, 1.05)	0.031
SNP_FokI			
Ff + ff*	1		
FF	0.45	(0.14, 1.49)	0.191
Etiology			
alcohol*	1		
viral	3.15	(0.77, 12.86)	0.111
other	5.22	(0.81, 33.69)	0.082
Sex			
male*	1		
female	0.12	(0.03, 0.58)	0.008
VDBP			
per unit	0.98	(0.97, 0.99)	<0.001
IL-8			
per unit	1.01	(1.00, 1.02)	0.004
LBP			
per unit	1.03	(1.01, 1.06)	0.011

*Reference category.

Abbreviations: OR, odds ratio; C.I., confidence interval; SNP, single nucleotide polymorphism; VDBP, vitamin D binding protein; LBP, lipopolysaccharide binding protein.

### *VDR* polymorphisms and the etiology of liver cirrhosis

The grouping of cirrhotic population according to disease etiology was performed as follows: patients with cirrhosis of viral origin (n = 40, 44.2%), alcoholic origin (n = 32, 36.4%) and other etiologies (n = 17, 19.4%). None of the *VDR* polymorphisms interacted significantly with the etiology of the disease, indicating that the effect of the polymorphisms is similar across all groups regarding cirrhosis’ etiology.

### Association between vitamin D and VDBP levels with VDR polymorphisms

We found no statistically significant differences between serum 25(OH) vitamin D levels and VDBP levels in relation to *VDR* polymorphisms.

### Linkage disequilibrium of *VDR* polymorphisms in cirrhotic patients

**Figure 1:**
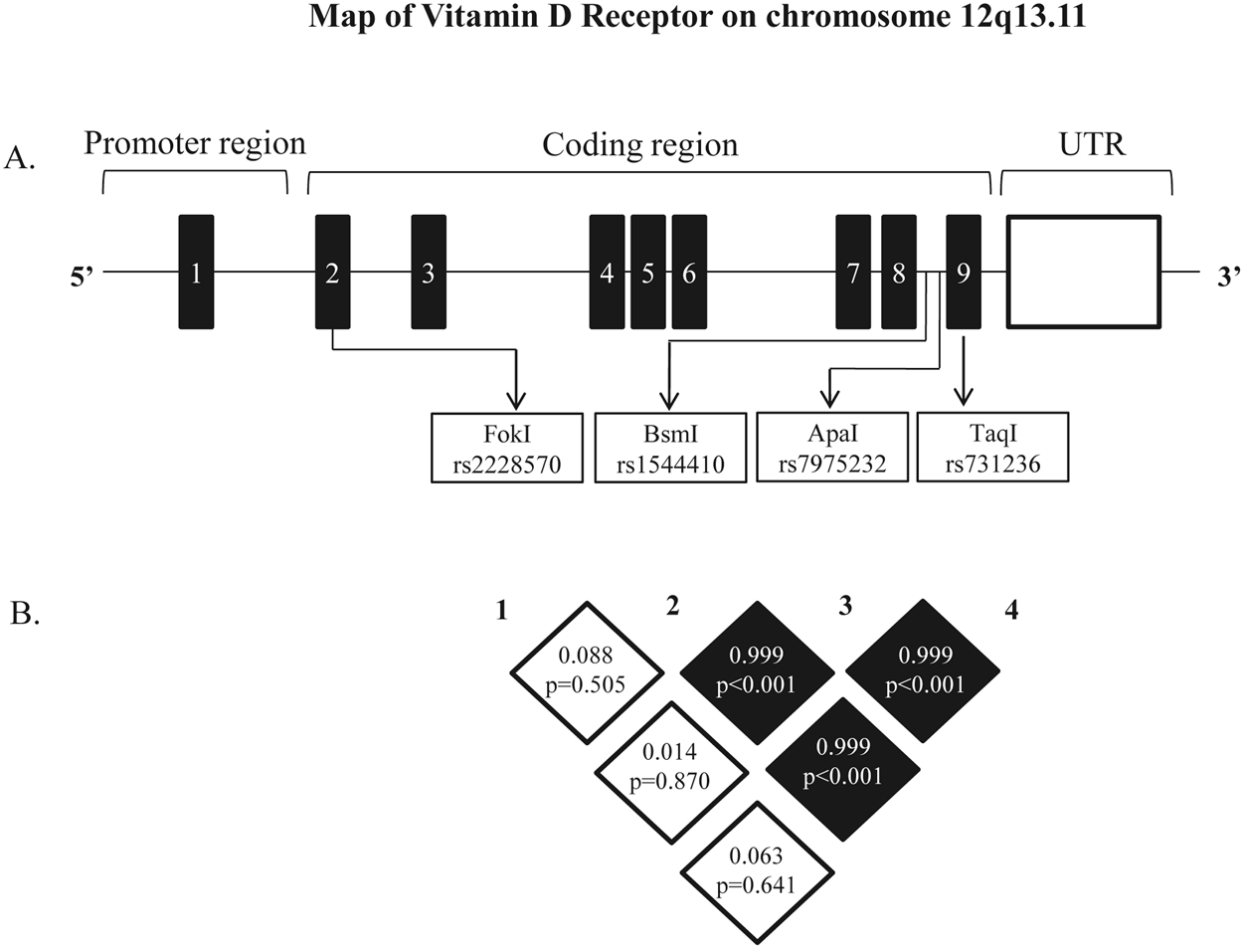
(**A**) Structure of genomic region of the *VDR* gene on chromosome 12q13.11. (**A**) The black boxes demonstrate the exons of the *VDR* gene. The approximate locations of the examined polymorphisms are indicated by arrows. (**B**) Schematic representation of pairwise linkage disequilibrium (LD) pattern. (**A**) LD pattern of the *VDR* gene polymorphisms [BsmI A > G (B > b), ApaI A > C (A > a), TaqI C > T (T > t) and FokI C > T (F > f)] in the studied population (n = 89). Each square represents the D’ values and the p values between the pairs of polymorphisms. The intensity of the dark color of the blocks is proportional to the D’ value, indicating the strength LD between polymorphisms. Black boxes, high LD; white boxes, low LD.

Linkage disequilibrium analysis revealed very strong LD between BsmI and TaqI (D’ = 0.999), BsmI and ApaI (D’ = 0.999) and TaqI and ApaI (D’ = 0.999) polymorphisms. In contrast, very weak LD was detected between FokI and BsmI (D’ = 0.088), FokI and TaqI (D’ = 0.063), FokI and ApaI (D’ = 0.014) polymorphisms (Fig. 1).

### Haplotype analysis of VDR polymorphisms in relation to disease severity

Haplotype association with cirrhosis severity was evaluated by the distribution of VDR haplotypes in the different CP stages. Estimated VDR haplotype frequencies of FokI, BsmI, ApaI and TaqI polymorphisms are reported in Table 6. The results showed that in patients with CP stage C, BAT haplotype was more frequent suggesting a potential increased risk for advanced cirrhosis, whereas the complementary haplotype bat was more common in patients with CP stage A; however, this difference was no significant (LR test *p* = 0. 581).

**Table 6:** Haplotype frequencies and association with cirrhosis severity.

Haplotypes	CP stage					
	A		B		C	
	n	Frequency (%)	n	Frequency (%)	n	Frequency (%)
BAT	35	19.7	15	8.5	20	11.2
bat	37	20.8	24	13.5	7	3.9
bAt	18	10.1	10	5.6	10	5.6
BAt	0	0.0	1	0.01	1	0.01
baT	0	0.0	0	0.0	0	0.0
bAT	0	0.0	0	0.0	0	0.0
Bat	0	0.0	0	0.0	0	0.0
BaT	0	0.0	0	0.0	0	0.0

Abbreviations: CP stage, Child-Pugh stage.

### Survival analysis

The cumulative mortality rate was 31.46% (28 out of 89 patients), after a median follow-up of 16 months (IQR: 3–40 months). The main causes of mortality were liver failure (n = 19, 67.9%), HCC (n = 4, 14.3%), renal failure (n = 3, 10.7%), bleeding (n = 1, 3.6%) and other causes (n = 1, 3.6%). In the univariable Cox regression analysis, the following factors were found to be significantly associated with mortality: CP score (*p* < 0.001), MELD (*p* < 0.001), VDBP levels (*p* = 0.003), IL-6 (*p* = 0.016), IL-8 (*p* = 0.001), LBP levels (*p* = 0.020) and the CP stage III (*p* < 0.001). In the multivariate analysis, CP score [HR: 1.26, 95% CI (1.02–1.56) *p* = 0.035], MELD [HR: 1.15, 95% CI (1.03–1.28) *p* = 0.012] and the presence of FF genotype [ff genotype vs FF: HR = 0.22 95% CI (0.06–0.77), *p* = 0.018)] were significant independent prognostic factors for patient survival (Table 7).

**Table 7:** Univariate and multivariate cox regression analyses for cirrhotic patients’ survival.

*Factor*	*HR*	95% C.I.	*p*-value
Univariate analysis			
**CP score**			
per unit	1.47	(1.26, 1.71)	**<0.001**
**MELD**			
per unit	1.20	(1.11, 1.29)	**<0.001**
**VDBP**			
per unit	0.99	(0.99, 1.00)	**0.003**
**IL-12**			
per unit	1.02	(0.98, 1.06)	0.287
**TNF-a**			
per unit	1.04	(0.94, 1.17)	0.440
**IL-1β**			
per unit	1.00	(0.97, 1.04)	0.827
**IL-6**			
per unit	1.00	(1.00, 1.00)	**0.016**
**IL-8**			
per unit	1.00	(1.00, 1.00)	**0.001**
**IL-10**			
per unit	1.01	(0.98, 1.04)	0.455
**LBP**			
per unit	1.02	(1.00, 1.04)	**0.020**
**Age**			
per unit	1.01	(0.98, 1.05)	0.438
**Sex**			
Male*	1		
Female	1.16	(0.50, 2.71)	0.735
**CP stage**			
A*	1		
B	2.87	(0.99, 8.33)	0.052
C	9.06	(3.19, 25.76)	**<0.001**
**SNP_BsmI**			
bb*	1		
Bb	0.55	(0.24, 1.30)	0.175
BB	1.81	(0.67, 4.86)	0.242
**SNP_TaqI**			
tt*	1		
Tt	0.60	(0.26, 1.42)	0.249
TT	1.92	(0.71, 5.18)	0.195
**SNP_FokI**			
ff*	1		
Ff	0.72	(0.26, 2.03)	0.538
FF	0.51	(0.18, 1.46)	0.210
**SNP_ApaI**			
aa*	1		
Aa	1.21	(0.27, 5.41)	0.803
AA	2.11	(0.48, 9.21)	0.323
***Factor***	***HR***	**95% C.I.**	***p*** **-** **value**
**Multivariate analysis**			
**CP score**			
per unit	1.26	(1.02, 1.56)	**0.035**
**MELD**			
per unit	1.15	(1.03, 1.28)	**0.012**
**SNP_FokI**			
ff*	1		
Ff	0.37	(0.12, 1.13)	0.080
FF	0.22	(0.06, 0.77)	**0.018**

*Reference category.

Abbreviations: HR, hazard ratio; C.I., confidence interval; CP, child pugh; MELD, model for end-stage liver disease; VDBP, vitamin D binding protein; LBP, lipopolysaccharide binding protein; SNP, single nucleotide polymorphism.

## Discussion

This is the first report of an association between polymorphisms of the *VDR* gene and cytokine levels, severity of liver disease and survival in patients with liver cirrhosis. In particular, an independent association between BsmI, ApaI, and TaqI *VDR* polymorphisms and the severity of liver cirrhosis is clearly shown. Moreover, the presence of FF genotype of FokI polymorphism is associated with a better prognosis regarding survival in this cohort. These features appear to be independent of the etiology of liver cirrhosis, as they observed in patients of any cause.

Vitamin D promotes the stimulation of innate immunity, the differentiation of monocytes, the inhibition of lymphocyte proliferation and cytokine secretion by T and B cells^22,23^. *VDR* acts as a ligand-stimulated transcription factor and activates 1,25(OH)_2_D_3_ at the transcriptional level. The activation of *VDR* contributes to the regulation of immune response by inhibiting T helper 1 (Th1) cell proliferation and pro-inflammatory cytokine production and inducing Th2 cell proliferation and anti-inflammatory cytokine production^7,22–26^. The presence of *VDR* polymorphisms possibly leads to a dysfunctional receptor, affecting VDR activity and the subsequent vitamin D-mediated effects^26^.

The association between *VDR* polymorphisms and the occurrence of chronic liver disease from different etiologies such as autoimmune hepatitis, PBC, HCC or HBV infection has been investigated with conflicting results^8,10–16,27^. Previous reports have identified gene polymorphisms which affect the progression of liver fibrosis^28–31^. The relationship between liver fibrosis progression and the presence of *VDR* polymorphisms (ApaI, TaqI and BsmI) has been investigated, indicating that in PBC patients, BsmI and TaqI were associated with progressive cirrhosis^10^ and in NAFLD patients, *VDR* mRNA expression and profibrogenic genes were significantly affected by BsmI polymorphism^18^. The effect of bAt haplotype in fibrosis progression has been investigated in HCV patients as well, giving conflicting results^18,19,32^. Our results indicate that the presence of ApaI polymorphism (AA genotype) is related to significant higher levels of IL-1β and IL-8. The increased levels of these pro-inflammatory cytokines suggest that the ApaI *VDR* polymorphism leads to a less active VDR protein which may contribute to a disturbance of Th1/Th2 balance, a transition to Th1 polarization and a decreased activity of vitamin D-related signaling pathways.

**Figure 2:**
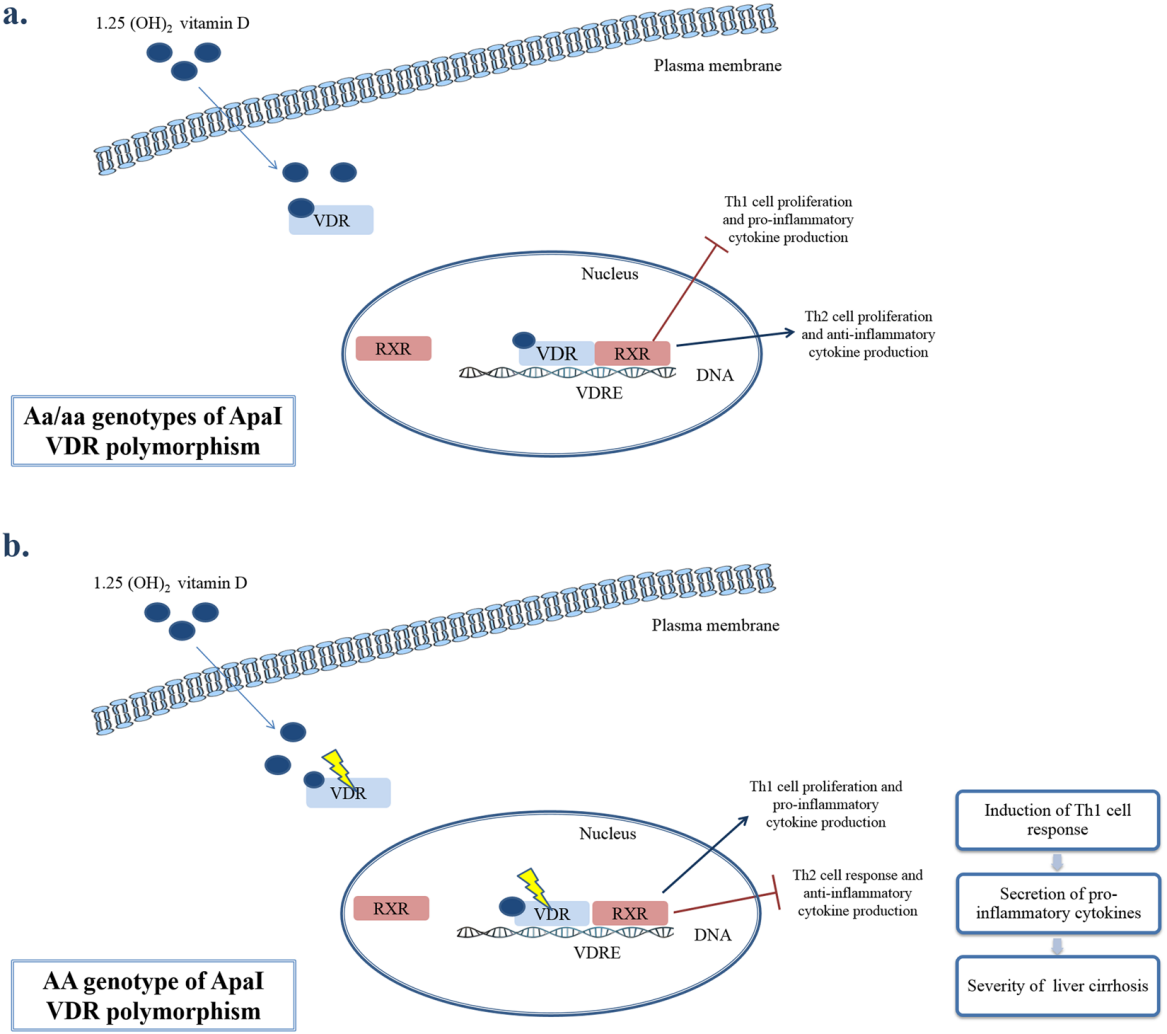
Schematic representation illustrating our proposed mechanism of how ApaI *VDR* polymorphisms potentially affect the progression of liver cirrhosis. (**a**) Presence of Aa/aa genotypes of ApaI *VDR* polymorphism. *VDR* is an intracellular ligand-activated transcription factor that specifically binds 1,25(OH)2D_3_ and regulates the expression of several target genes. Upon the activation of vitamin D, the ligated VDR heterodimerizes with retinoid X receptor (RXR) which is necessary for DNA binding, translocates to the nucleus, binds to vitamin D response elements (VDRE) and recruits other nuclear proteins to the transcriptional pre-initiation complex. This process results in the transcriptional activation or suppression of the target genes through the interaction with nuclear receptor co-activators or co-repressors. The binding of VDR with vitamin D may modulate cytokine responses by T cells, inhibiting Th1 cell proliferation and pro-inflammatory cytokine secretion and activating Th2 cell proliferation and anti-inflammatory cytokine secretion. (**b**) Presence of AA genotype of ApaI *VDR* polymorphism. The presence of polymorphisms may impair the activity of the *VDR* resulting in a dysfunctional receptor. The dimerization of the 1,25(OH)2D_3_-VDR with RXR may be hindered by the existence of genetic variations thus affecting VDR activity and subsequent downstream vitamin D-mediated effects. This impaired process may lead to disturbance of the Th1/Th2 balance, resulting in a transition to Th1 cell response and pro-inflammatory cytokine secretion that is closely related the progression of liver cirrhosis.

Several studies have demonstrated a positive correlation between higher pro-inflammatory cytokine levels and the severity of liver disease^33–37^. In this study, we have shown that the AA genotype of the ApaI polymorphism is related to decreased levels of platelets and increased levels of LBP, which are consistent with the progression of cirrhosis and portal hypertension development^38,39^. The presence of ApaI, TaqI and BsmI *VDR* polymorphisms could impede the interaction between vitamin D and VDR, resulting in ineffective vitamin D-VDR complex, impaired VDR-mediated transcription, decreased activity of vitamin D related signaling pathways, transition to a Th1 polarization, and consequently, to a more progressive form of liver cirrhosis (Fig. 2).

A second novel finding of this study, is the inverse association between the FokI polymorphism, particularly the FF genotype, with mortality in liver cirrhotic patients, imparting a protective role of this genotype in cirrhosis. The FokI polymorphism is located in the coding region of the *VDR* gene and results in a VDR protein with a different structure, creating a new start codon and consequently a VDR protein shortened by three amino acids^38,40^. This protein is more functional and has higher transcriptional activity compared to the long-length VDR protein^38,40,41^. FokI is the only polymorphism that was not associated with severity of liver cirrhosis in our study. The length of the VDR protein influences the regulation of gene transcription through occupation of recognition sites of other transcription factors and interference with their signaling pathways^7^. Therefore, a longer VDR protein may lead to a decreased transcriptional activity and an increased risk of susceptibility to disease^40^. These observations are in line with our study as we have shown that the presence of FokI polymorphism (FF genotype) is associated with significantly lower levels of IL-1β. Patients with the FF genotype produce a shorter form of VDR, leading to higher transcriptional activity, formation of more active complexes of VDR-vitamin D, inhibition of the Th1 response and induction of the Th2 cell response. Hence, patients with FokI FF genotype may have a better response to vitamin D resulting in a lower progression rate of cirrhosis. However, due to the fact that this hypothesis is of high interest, we suggest that it should be further explored in larger and more specific cohorts with more patients harboring the FokI polymorphism in order to be confirmed.

We have also shown the existence of strong linkage disequilibrium between the BsmI, ApaI and TaqI polymorphic sites in our cirrhotic population. These results are in agreement with previous reports suggesting an extensive LD between these genetic markers^10,14^. As these polymorphisms are in strong LD, it can be assumed that these single nucleotide polymorphisms (SNPs) contribute to the severity of cirrhosis in a dependent manner. Nevertheless, as these polymorphisms do not cause a functional change in the *VDR* gene, it is possible that BsmI, TaqI and ApaI are possibly genetic markers of other functional variations of the *VDR* gene or in other closely linked genes that are in linkage disequilibrium with the identified polymorphisms.

Some limitations of the current study should be acknowledged. The first limitation concerns the relatively small sample size, however our results are consistent with the reports on the association between *VDR* polymorphisms and the susceptibility to liver fibrosis^10,18,19^. Secondly, our study was performed on Caucasians patients and it would be interesting to perform the same analysis in different ethnic groups. Lastly, the single measurement of 25(OH)D at baseline may not be representative of the respective concentrations over time. However, there are reports supporting that although 25(OH)D levels present seasonal fluctuation, its levels remain stable over time^42,43^.

In conclusion, our results indicate that *VDR* polymorphisms are independently associated with the severity of liver cirrhosis and the survival of patients with liver disease, regardless of disease etiology, suggesting a potential influence of them in disease progression. Based on these results future studies will delineate causation between specific *VDR* polymorphisms and outcome/severity of liver cirrhosis, and the importance of *VDR* polymorphism analysis in clinical practice to identify patients at greater risk of disease progression and to modify patients’ surveillance and treatment accordingly.

## Methods

### Study design and participants

This study was a prospective cohort study, on 89 consecutive Caucasian patients with liver cirrhosis. During the recruitment, all cirrhotic patients were in stable clinical condition, without any severe complication of liver disease including gastro-intestinal bleeding, hepatorenal syndrome, moderate to severe hepatic encephalopathy, spontaneous bacterial peritonitis, malignancy, or organ failure. Patients with indications or history of bacterial infection at last 4 weeks prior to recruitment in the study, human immunodeficiency virus (HIV) infection and severe cardiopulmonary disease or renal failure were excluded. Severity of cirrhosis was assessed by the CP stage and the MELD score^44^. Diagnosis of cirrhosis was based on histological or compatible clinical, laboratory and imaging data^45–47^. After baseline examination, patients were followed in the hepatology clinic at regular intervals according to current guidelines^48^ until death, liver transplantation or completion of the study. The recruitment of the patients was performed at the University Hospital of Patras (Patras, Greece) between September 2009 and April 2013. Blood samples from all patients were collected throughout the year. Seasonal variability was defined as winter/spring from December to April and summer/autumn from May to October^49^. Sampling occurred mostly in winter/spring (70%) compared to summer/autumn (30%). All study participants, or their legal guardian, provided informed written consent prior to study enrollment. The study protocol was approved by Patras University Hospital Scientific Review Board and Ethics Committee. The Hospital abides by the 1975 Helsinki declaration on ethical principles for medical research involving human subjects. Further all the experiments were performed in accordance with relevant guidelines and regulations of the concerned ethical committee.

### Vitamin D assay

Serum samples were collected from the patients and stored at −80 °C until analysis. Serum 25(OH)D levels were determined using a 25(OH)D vitamin D ELISA kit for serum and plasma (Enzo Life Sciences, NY, USA), according to the manufacturer’s instructions. Currently accepted standards for the definition of Vitamin D status are: optimal vitamin D levels ≥30 ng/mL, vitamin D deficiency ≤20 ng/mL and vitamin D insufficiency between 20 and 30 ng/mL^50,51^.

### VDBP and LBP assays

Serum VDBP levels were determined using a human Vitamin D BP Quantikine ELISA Kit (R&D Systems, Minneapolis, MN, USA), and serum LBP levels using a human LBP ELISA kit (SunRed Biological Technology, Shanghai). Data analysis was performed using the Curve Expert 1.4 software.

### Cytokine Assays

Serum interleukin-12 **(**IL-12), IL-1β, IL-6, IL-8, IL-10 and tumor necrosis factor alpha (TNF-a) levels were determined using a Cytometric Bead Array (CBA) assay (Human Inflammatory Cytokines Kit, BD Biosciences, San Diego, CA, USA) run on a BD FACS Array Bioanalyzer. Data were analyzed using the FlowJo V7.5 software (Tree Star Inc., Ashland, OR, USA).

### DNA extraction

Genomic DNA was extracted using the NucleoSpin® Blood QuickPure kit (Macherey-Nagel, Germany). The DNA concentration of the samples was determined using a Nanodrop spectrophotometer (UV spectrophotometer Q3000, Quawell Technology, Inc., USA).

### *VDR* Genotyping

Genotyping was carried out using TaqMan SNP Genotyping Assays (Applied Biosystems; Foster City, USA). The PCR reactions were carried out in MicroAmp® Fast Optical 96-Well Reaction Plates (Applied Biosystems) on the Step One Plus real-time PCR system (Applied Biosystems, CA, USA). The rs731236 (TaqI), rs1544410 (BsmI), rs7975232 (ApaI) and rs2228570 (FokI) probes were designed using TaqMan pre-designed SNP genotyping assays (Applied Biosystems). Two non-template-control wells were included on each plate. DNA amplification was performed as follows: 95 °C for 10 min, followed by 40 cycles of 92 °C for 15 sec and 60 °C for 1 min.

### Statistical analysis

Continuous variables were summarized as medians and interquartile ranges (IQRs) while counts and corresponding percentages were calculated for categorical variables. All comparisons were performed using non-parametric tests: Fisher’s exact tests in case of frequencies’ comparisons, Mann-Whitney and Kruskal-Wallis tests for the comparison of median values between two groups and more than two groups, respectively. Correlations between vitamin D and VDBP levels with *VDR* polymorphisms were assessed by the Spearman’s coefficient. Multivariable ordinal logistic regression models were fitted, to test the hypothesis that the *VDR* polymorphisms are associated with the CP stage. Further analysis was conducted to explore whether these polymorphisms’ effect interacts with the etiology of cirrhosis, i.e. whether the effect of the polymorphisms is different in the subgroups of viral, alcoholic or other etiology’s cirrhosis. The *VDR* gene polymorphisms’ Hardy-Weinberg equilibrium was examined by means of chi square test goodness of fit test, i.e by comparing observed and expected count in each of the polymorphisms groups (wt/wt, mt/wt, mt/mt). Pairwise linkage disequilibrium (LD) analysis between the *VDR* gene polymorphisms was performed using the genetics package of R software. Allelic frequencies were estimated by the hapipf stata command, based on the expectation-maximization (EM) algorithm. The hypothesis of allelic association with the CP stage was tested using the likelihood-ratio (LR) test. Time to death was analyzed using the Cox survival model. Before fitting the models, the proportional hazards assumption was assessed for all variables based on Schoenfeld residuals. Individuals’ baseline clinical and laboratory variables, including the *VDR* polymorphisms, were considered as potential risk factors. For all models selection, the Collett’s approach was followed^52^. More specifically, all variables with a *p*-value < 0.200 were initially included and then eliminated using backwards selection. When a model that included only significant covariates was reached, variables initially excluded entered the final model one by one and tested for significance in the presence of already included significant variables. Analysis was performed using Stata 13.1 (StataCorp LP, College Station, Texas, USA). Level of significance α was set at 0.05.

## References

[1] Schuppan D, Afdhal NH (2008). Liver cirrhosis. Lancet..

[2] Sivanathan V (2014). Etiology and complications of liver cirrhosis: data from a German centre. Dtsch Med Wochenschr..

[3] Bataller R, North KE, Brenner DA (2003). Genetic polymorphisms and the progression of liver fibrosis: a critical appraisal. Hepatology..

[4] Bhalla AK, Amento EP, Clemens TL, Holick MF, Krane SM (1983). Specific high-affinity receptors for 1,25-dihydroxyvitamin D3 in human peripheral blood mononuclear cells: presence in monocytes and induction in T lymphocytes following activation. J Clin Endocrinol Metab..

[5] Kato S (2000). The function of vitamin D receptor in vitamin D action. J Biochem.

[6] Carlberg C, Campbell MJ (2013). Vitamin D receptor signaling mechanisms: integrated actions of a well-defined transcription factor. Steroids..

[7] van Etten E (2007). The vitamin D receptor gene FokI polymorphism: functional impact on the immune system. Eur J Immunol..

[8] Vogel A, Strassburg CP, Manns MP (2002). Genetic association of vitamin D receptor polymorphisms with primary biliary cirrhosis and autoimmune hepatitis. Hepatology..

[9] Wang X, Cheng W, Ma Y, Zhu J (2017). Vitamin D receptor gene FokI but not TaqI, ApaI, BsmI polymorphism is associated with Hashimoto’s thyroiditis: a meta-analysis. Sci Rep..

[10] Kempinska-Podhorecka A (2012). Vitamin d receptor polymorphisms predispose to primary biliary cirrhosis and severity of the disease in polish population. Gastroenterol Res Pract..

[11] Halmos B (2000). Association of primary biliary cirrhosis with vitamin D receptor BsmI genotype polymorphism in a Hungarian population. Dig Dis Sci..

[12] Lakatos LP (2002). Vitamin D receptor, oestrogen receptor-alpha gene and interleukin-1 receptor antagonist gene polymorphisms in Hungarian patients with primary biliary cirrhosis. Eur J Gastroenterol Hepatol..

[13] Tanaka A (2009). Vitamin D receptor polymorphisms are associated with increased susceptibility to primary biliary cirrhosis in Japanese and Italian populations. J Hepatol..

[14] Falleti E (2010). Vitamin D receptor gene polymorphisms and hepatocellular carcinoma in alcoholic cirrhosis. World J Gastroenterol..

[15] Yao X (2013). The associated ion between the VDR gene polymorphisms and susceptibility to hepatocellular carcinoma and the clinicopathological features in subjects infected with HBV. Biomed Res Int..

[16] Suneetha PV (2006). Association between vitamin D receptor, CCR5, TNF-alpha and TNF-beta gene polymorphisms and HBV infection and severity of liver disease. J Hepatol..

[17] Fan L (2005). Genetic association of vitamin D receptor polymorphisms with autoimmune hepatitis and primary biliary cirrhosis in the Chinese. J Gastroenterol Hepatol..

[18] Baur K (2012). Combined effect of 25-OH vitamin D plasma levels and genetic vitamin D receptor (NR 1I1) variants on fibrosis progression rate in HCV patients. Liver Int..

[19] Beilfuss A (2015). Vitamin D counteracts fibrogenic TGF-beta signalling in human hepatic stellate cells both receptor-dependently and independently. Gut.

[20] Tilg H (2001). Cytokines and liver diseases. Can J Gastroenterol..

[21] Zhou WC, Zhang QB, Qiao L (2014). Pathogenesis of liver cirrhosis. World J Gastroenterol..

[22] Lemire JM, Archer DC, Beck L, Spiegelberg HL (1995). Immunosuppressive actions of 1,25-dihydroxyvitaminD3: preferential inhibition of Th1 functions. J Nutr..

[23] Willheim M (1999). Regulatory effects of 1alpha,25-dihydroxyvitamin D3 on the cytokine production of human peripheral blood lymphocytes. J Clin Endocrinol Metab..

[24] Penna G, Adorini L (2000). 1 Alpha,25-dihydroxyvitamin D3 inhibits differentiation, maturation, activation, and survival of dendritic cells leading to impaired alloreactive T cell activation. J Immunol..

[25] Valdivielso JM, Fernandez E (2006). Vitamin D receptor polymorphisms and diseases. Clin Chim Acta..

[26] Cao Y, Wang X, Cao Z, Cheng X (2015). Association of Vitamin D receptor gene TaqI polymorphisms with tuberculosis susceptibility: a meta-analysis. Int J Clin Exp Med..

[27] Fan LY (2005). Genetic association of cytokines polymorphisms with autoimmune hepatitis and primary biliary cirrhosis in the Chinese. World J Gastroenterol..

[28] Uygun A (2017). The association of nonalcoholic fatty liver disease with genetic polymorphisms: a multicenter study. Eur J Gastroenterol Hepatol..

[29] Joshita S, Umemura T, Tanaka E, Ota M (2017). Genetic Contribution to the Pathogenesis of Primary Biliary Cholangitis. J Immunol Res..

[30] Motawi T, Shaker OG, Hussein RM, Houssen M (2016). Polymorphisms of alpha1-antitrypsin and Interleukin-6 genes and the progression of hepatic cirrhosis in patients with a hepatitis C virus infection. Balkan J Med Genet..

[31] Medrano LM, Jimenez-Sousa MA, Fernandez-Rodriguez A, Resino S (2017). Genetic Polymorphisms Associated with Liver Disease Progression in HIV/HCV-Coinfected Patients. AIDS Rev..

[32] de Azevedo LA, Matte U, da Silveira TR, Alvares-da-Silva MR (2017). Genetic variants underlying vitamin D metabolism and VDR*-*TGFbeta-1-SMAD3 interaction may impact on HCV progression: a study based on dbGaP data from the HALT-C study. J Hum Genet..

[33] An L, Wang X, Cederbaum AI (2012). Cytokines in alcoholic liver disease. Arch Toxicol..

[34] Giron-Gonzalez JA (2004). Implication of inflammation-related cytokines in the natural history of liver cirrhosis. Liver Int.l.

[35] Goral V, Atayan Y, Kaplan A (2011). The relation between pathogenesis of liver cirrhosis, hepatic encephalopathy and serum cytokine levels: what is the role of tumor necrosis factor alpha?. Hepatogastroenterology..

[36] Kawaratani H (2013). The effect of inflammatory cytokines in alcoholic liver disease. Mediators Inflamm..

[37] Li CP (1996). Plasma interleukin-8 levels in patients with post-hepatitic cirrhosis: relationship to severity of liver disease, portal hypertension and hyperdynamic circulation. J Gastroenterol Hepatol..

[38] Alimirah F, Peng X, Murillo G, Mehta RG (2011). Functional significance of vitamin D receptor FokI polymorphism in human breast cancer cells. PloS one..

[39] Reiberger T (2013). Non-selective betablocker therapy decreases intestinal permeability and serum levels of LBP and IL-6 in patients with cirrhosis. J Hepatol..

[40] Whitfield GK (2001). Functionally relevant polymorphisms in the human nuclear vitamin D receptor gene. Mol Cell Endocrinol..

[41] Elhoseiny SM, Morgan DS, Rabie AM, Bishay ST (2016). Vitamin D Receptor (VDR) Gene Polymorphisms (FokI, BsmI) and their Relation to Vitamin D Status in Pediatrics betaeta Thalassemia Major. Indian J Hematol Blood Transfus..

[42] Jorde R (2010). Tracking of serum 25-hydroxyvitamin D levels during 14 years in a population-based study and during 12 months in an intervention study. Am J Epidemiol..

[43] Sonderman JS, Munro HM, Blot WJ, Signorello LB (2012). Reproducibility of serum 25-hydroxyvitamin d and vitamin D-binding protein levels over time in a prospective cohort study of black and white adults. Am J Epidemiol..

[44] Chen K, Cao X, Zheng Y, Xu M, Peng J (2014). Comparative study of the MELD-Na and Child-Turcotte-Pugh scores as short-term prognostic indicators of acute-on-chronic hepatitis B liver failure. Zhonghua Gan Zang Bing Za Zhi..

[45] Guan R, Lui HF (2011). Treatment of hepatitis B in decompensated liver cirrhosis. Int J Hepatol..

[46] Strauss E, Dias Teixeira MC (2006). Quality of life in hepatitis C. Liver Int..

[47] Gutteling JJ, de Man RA, Busschbach JJ, Darlington AS (2007). Overview of research on health-related quality of life in patients with chronic liver disease. Neth J Med..

[48] Garcia-Tsao G, Lim JK (2009). Management and treatment of patients with cirrhosis and portal hypertension: recommendations from the Department of Veterans Affairs Hepatitis C Resource Center Program and the National Hepatitis C Program. Am J Gastroenterol..

[49] Webb AR, Kline L, Holick MF (1988). Influence of season and latitude on the cutaneous synthesis of vitamin D3: exposure to winter sunlight in Boston and Edmonton will not promote vitamin D3 synthesis in human skin. J Clin Endocrinol Metab..

[50] Wittnich C, Belanger MP, Askin N, Boscarino C, Wallen WJ (2004). Lower liver transplant success in females: gender differences in metabolic response to global ischemia. Transplant Proc..

[51] Konstantakis C, Tselekouni P, Kalafateli M, Triantos C (2016). Vitamin D deficiency in patients with liver cirrhosis. Ann Gastroenterol..

[52] Collett, D. *Modelling survival data in medical research*. (CRC press, 2015).

